# Effect of a curriculum transformation on pharmacy student self-efficacy, self-reported activities, and satisfaction in degree and career choice

**DOI:** 10.1186/s12909-023-04280-7

**Published:** 2023-05-02

**Authors:** Harjit Kaur Singh, Kayley Marie Lyons, Tina Penick Brock, Daniel Thomas Malone

**Affiliations:** 1 Faculty of Pharmacy and Pharmaceutical Sciences, Monash University, Parkville, VIC Australia; 2 Centre for Digital Transformation of Health, University of Melbourne, Melbourne, Australia; 3 University of Colorado Skaggs School of Pharmacy & Pharmaceutical Sciences, Melbourne, U.S.; 4 Faculty of Pharmacy and Pharmaceutical Sciences, 407 Royal Parade, Parkville, VIC 3052 Australia

**Keywords:** Pharmacy, Education, Survey, Clinical confidence, Student satisfaction, Curriculum transformation

## Abstract

**Background:**

Curriculum revision in healthcare programs occurs frequently, but to undergo a whole degree transformation is less common. Also, the outcomes of curriculum redesign interventions on the selfreported clinical decision making, experiences, and perceptions of graduates of health education programs is unclear. This study evaluated these factors as an outcome of a pharmacy degree whole-curriculum transformation.

**Methods:**

A 25-item cross-sectional end-of-course survey was developed to evaluate pharmacy student decisions, experiences, and perceptions upon completion of degree, pre- and post- curriculum transformation. A two-way analysis of variance (ANOVA) was used to determine whether the responses to the items classed within the main factors differed across the two cohorts. Independent t-tests were used to examine the student responses to the individual questions between the two cohorts.

**Results:**

Graduates from the transformed degree had greater self-efficacy in clinical activities, were more satisfied with their education, found course activities more useful, and were more confident in their career choice. Transformed pharmacy degree students also reported spending more time on weekdays and weekends on activities such as attending lectures and working. Student satisfaction with their choice to attend pharmacy school was also significantly higher in transformed degree students.

**Conclusions:**

Responses to the end of degree survey indicate that students who completed the transformed pharmacy curriculum have had positive experiences throughout their degree and felt more prepared for practice as pharmacists in comparison to students who completed the established degree. These results add value to those collected from other sources (e.g., student evaluations, assessment scores, preceptors focus groups, and other stakeholder inputs) consistent with a comprehensive quality improvement model.

**Supplementary Information:**

The online version contains supplementary material available at 10.1186/s12909-023-04280-7.

## Background

Health professional training programs have a societal obligation to produce graduates that are capable practitioners with skills needed for lifelong professional development. Consequently, programs must undergo regular review, evaluation and revision to ensure they are fit-for-purpose in the changing landscape of healthcare needs. Pharmacy programs in Australia also must meet the Australian Qualifications Standards and to receive accreditation from the Australian Pharmacy Council (APC). Embracing these challenges, the Monash University Faculty of Pharmacy and Pharmaceutical Sciences introduced a transformed pharmacy degree in 2017. The process of developing this degree has been described elsewhere in detail. [1] Key changes between the former (termed “established”) degree and the transformed degree include an increase in the number of experiential placements that included entrustable professional activities (EPAs), [2, 3] and the introduction of a Master’s component with a consistent flipped classroom approach. This “DEAR” model consists of online preparatory material and related tasks termed “Discovery”, whole class interactive lectures termed “Explore”, small class team-based workshops termed “Apply”, and regular cycles of student reflection on the development of skills, through a structured skills coaching program with an e-portfolio, “close the loop” lectures and written reflections (“Reflect”). Furthermore, unlike the established degree that had a solid focus on basic sciences, particularly in the first two years of the degree, the transformed degree was designed with a core focus on pharmacotherapeutic applications with integrated research and interprofessional training. [3].

Traditionally, the outcomes of curriculum interventions have been evaluated by collecting information from multiple sources. These may include student evaluation of individual courses [4] or the student evaluation of both teaching and units referred to as student evaluation of teaching and units (SETU) at Monash University, [1] assessment results, [5] preceptor feedback, [3] graduation rates, [6] and employment rates after graduation. [6] These data sources are often organized using an outcomes framework such as the recently revised Kirkpatrick Model of Evaluation, [7] which is popular in health professions education. Investigating student perceptions at the end of a curriculum is a more constructive approach of analyzing impressions and overall curricular quality. [8–10] End-of-curriculum surveys have been used by medical schools to investigate student and graduate perceptions of curriculum. [11–13] These kinds of surveys have predominantly used Likert-type questions [12, 13] which are simple, specific, and ubiquitous, [14] Surveys that capture medical student experiences towards the end of their degree have identified individual courses that lack clinical relevance or are not perceived to be useful for clinical practice expectations. [13] This feedback has identified areas for curriculum revision. [12, 13] A disadvantage of these surveys however, is that they can often be quite long; including an extensive list of questions in order to address multiple areas of the degree. [11, 13, 15] Furthermore, limited research incorporating end-of-degree surveys means that benchmarking and adaptation to pharmacy education is limited. [15].

One pharmacy organization that has implemented a national (USA) graduating student survey broadly is the American Association of Colleges of Pharmacy (AACP). [16, 17] The AACP graduating student survey examines the views of new pharmacists and is conducted annually by most Pharmacy colleges in the US. It includes 80 questions, encompassing eight key areas based on Accreditation Council of Pharmacy Education (ACPE) 2016 Standards. [16] Data from the survey is managed by AACP and is used to track trends in the views of graduating student pharmacists. [16] Although very comprehensive, the survey is specific to the US pharmacy education standards. Differences in various aspects, such as jurisdiction, practice standards, health care needs and educational institutions, affect the transferability of the AACP graduating student survey to pharmacy schools globally. Likewise, other well-known surveys such as the Association of American Medical Colleges (AAMC) graduation questionnaire [11, 18, 19] specifically evaluate medical students’ perceptions and do not take into consideration the intricacies of pharmacy student education and experiences. Hence, there is a need for an Australian pharmacy education specific survey. In addition, although there have been reports of pharmacy schools previously undergoing partial or complete transformation, a lack of evaluation and dissemination of findings within the literature suggests a need for comprehensive and transparent research. Moreover, at times researchers have published studies about individual courses, despite changes being made at the degree level that otherwise invalidate evaluations that should have been scoped at the degree-level. Although course evaluations such as SETU [1] provide student feedback to faculty about teaching, course design and delivery about individual courses, [4] they are limited in their capacity to offer evaluations about a degree in its entirety. Effective components that should be considered when designing an end of degree pharmacy survey include question clarity, the number of questions, rating scales and the standardization of questions. [4].

In this paper, the design and implementation of an end of degree survey describing the outcomes of a curriculum transformation on student satisfaction, time of task, self-efficacy for pharmacy skills and perceptions for utility-value of teaching activities is described. The survey contributes to the limited body of work within this field and thus may be useful to other pharmacy schools undertaking degree transformations that are interested in investigating the outcomes of curriculum change on student perceptions and preparedness for practice.

## Methods

A cross-sectional survey design was used to examine the association between student characteristics, experiences, and perceived preparedness as pharmacists upon completion of the transformed Monash pharmacy degree in comparison to the established degree. A 25-item survey was constructed for this study based adaptations of the following surveys and scales that had sufficient evidence for validity and reliability: the AACP graduating student survey, [16] the Association of American Medical Colleges (AAMC) graduation questionnaire, [18] self-efficacy scales, [20, 21] utility-value belief scales [22] and time use scales. [23].

The scales were chosen explicitly to measure specific student characteristics as an outcome of complete degree transformation. Self-efficacy scales are a powerful proxy measure for future student performance. Theoretically, if students have higher self-efficacy, then their actual performance will be higher. [20] Therefore, the scale was considered an appropriate measure for examining confidence.

The utility-value beliefs scales measure student perceptions of activities or tasks, [24] the outcomes of which often decide what students persist in and the choices they make. The transformed degree curriculum aimed to produce graduates with diversity in their career choices and an increased interest in leadership and research activities, hence questions addressing student satisfaction and career choice were also included in the survey.

It was also important to evaluate time use, as the design of the transformed curriculum was aimed at being a more effective pedagogical model that included students committing the same amount of time to their studies as established degree students. Considerations were also made to reflect the Australian location and the desired outcomes of the transformed Monash pharmacy curriculum. The survey was designed to collect information on eight key factors: student demographics, self-efficacy in clinical activities, weekday time management, weekend time management, satisfaction in education, utility value of course activities, and self-efficacy in career choice (Appendix 1).

Reports of self-efficacy were collected on a scale out of 100, with higher scores indicating more favorable outcomes. Data for items contained within the factor ‘usefulness of course activities’ were collected on a 7-point Likert scale, while items contained within the factor ‘career choice scale’ were collected on a 5-point Likert scale, with higher scores indicating more favorable outcomes. To measure items contained in ‘weekday time management’ and ‘weekend time management’, students were asked to report the ‘time spent’ based on a 24-hour scale they had spent on various tasks.

### Ethics approval

for this study was received from the Monash University ethics committee (2019–21,702). The survey was distributed to fourth-year students from the established Monash Pharmacy degree in late October 2019 in a break during a required workshop. In 2020 due to the COVID-19 restrictions the survey was distributed online using Qualtrics® to the fourth-year students from the transformed degree. Upon completion of the survey all students received a $50 AUD gift card.

### Data Analysis

Survey responses were analyzed using GraphPad Prism 9.3.1 with the significance level set at *p* < 0.05. Descriptive analysis was carried out on survey scores obtained from the transformed degree and established degree cohorts, using mean and standard deviation (SD) for quantifiable variables. Chi- square tests were used to examine the association between the cohort and the various demographic questions related to students’ country of birth, language spoken as a child, language spoken at home, internship plans and degree entry mode. A two-way analysis of variance (ANOVA) was used to determine whether the student responses to the items classed within the main factors differed across the two cohorts. Independent *t*-tests were used to examine the student responses to the individual questions between the two cohorts.

## Results

Completed questionnaires were obtained from 237 to 280 4th year pharmacy students from the established degree in 2019 (response rate 84.64%) and 146 of 204 4th year pharmacy students from the transformed degree in 2020 (response rate 71.56%).

Table 1 shows the demographic information of pharmacy students who completed the end of degree survey. There was a greater proportion of students who were graduate entry students in the established degree (21% of students in the cohort) compared to the established degree (16% of students in the cohort), however this difference was not statistically significant (p = 0.24). Graduate entry students are those students that had completed a science or biomedical science degree (or equivalent), and then completed two intensive courses in their first year of their pharmacy degree in the same year they completed courses that third year students completed. The proportion of students born overseas or in Australia between the two degrees was similar, as was the proportion of languages first learned as a child and languages spoken at home. Intrinsically, the chi- square test also showed that there were no significant differences between the two cohorts in all categories except ‘plans for next year’ (p < 0.05), whereby a greater percentage of students from the transformed degree (44.70%) compared to the established degree (26.90%) indicated that they plan on undertaking a hospital internship. The proportion of students planning on undertaking a community internship the following year was higher in the established degree, 64.50% compared with students in the transformed degree 48.70%. The proportion of students unsure about their plans for the following year was 6.70% in the transformed degree to compared to 8.50% in the established degree.

**Table 1:** Demographics of pharmacy students who completed the survey

Variable		Established degree students n (%)	Transformed degree students n (%)	*χ*,^*2*^p*
Completed survey		237 (84.64%)	146 (71.56%)	
Graduate entry student	Yes	49 (21%)	24 (16%)	1.40, 0.24
Country of birth	Australia	103 (43.80%)	80 (53.70%)	3.56, 0.06
	Overseas	132 (56.20%)	69 (46.30%)	
Language first learned as a child	English	102 (43.60%)	72 (48.00%)	1.81, 0.40
	English + other	5 (2.10%)	1 (0.70%)	
	Other	127 (54.30%)	77 (53.10%)	
Language spoken at home	English	92 (39.10%)	61 (41.70%)	1.05, 0.60
	English + other	32 (13.60%)	25 (16.70%)	
	Other	111 (47.20%)	64 (42.70%)	
Plans for next year	Community internship	151 (64.50%)	73 (48.70%)	12.86, < 0.05
	Hospital internship	63 (26.90%)	67 (44.70%)	
	Unknown	20 (8.50%)	10 (6.70%)	
Country of internship	Australia	205 (86.90%)	137 (90.10%)	3.08, 0.38
	Overseas	5 (2.10%)	2 (1.30%)	
	Blank	22 (9.30%)	13 (8.60%)	
	Unknown	4 (1.70%)	0 (0%)	

*p-value related to chi- square

The two-way ANOVA results (Table 2) showed that transformed pharmacy degree students reported greater self-efficacy in clinical activities (F_1,8250_ = 134.6; *p* < 0.0001), were more satisfied with their education (F_1,1107_ = 113.1; *p* < 0.0001), found course activities more useful (F_1,2214_ = 10.32; *p* = 0.0013) and were more confident in their career choice (F_1,737_ = 4.86; *p* = 0.0278) than established degree students. Transformed pharmacy degree students also reported spending more time on weekdays (F_1,2590_ = 8.87; *p* = 0.0029), and weekends (F_1,2590_ = 17.12; *p* < 0.0001) on various activities such as attending lectures and working than established degree students.

**Table 2:** Two-way ANOVA results evaluating student responses from the established degree and the new degree

Factor	N items, Scale ^a^	*df*	SS	Mean square	F	*p* value*
Self-efficacy in clinical activities	22	1	30,146	30,146	134.60	*p* < 0.0001
Weekday time management	7	1	40.10	40.10	8.87	*p* = 0.0029
Weekend time management	7	1	79.76	79.76	17.12	*p* < 0.0001
Satisfaction in education	3	1	42,343	42,343	113.10	*p* < 0.0001
Usefulness of course activities	6, (Scale 1–7) ^a^	1	15.58	15.58	10.32	*p* = 0.0013
Self-efficacy in career choice	2 (Scale 1–5) ^a^	1	7.20	7.20	4.86	*p* = 0.0278

^a^ item coded such that higher scores indicate higher satisfaction

The independent sample *t-*tests (Table 3) showed that in comparison to students in the established degree, students in the transformed degree reported having greater self-efficacy in conducting a detailed and systematic medication history (Q11_2, p < 0.00001), reconciling medication history (Q11_3, p = 0.03), counselling on the use of commonly prescribed medications (Q11_5, p = 0.04), counselling on the use of devices (Q11_6, p < 0.001), writing comprehensive clinical notes (Q11_7, p < 0.01), assessing whether medication doses are appropriate (Q13_1, p < 0.000001), assessing whether labs/tests are in-range or out-of-range (Q13_4, p < 0.001), identifying medication related problems (Q13_3, p < 0.000001), using resource databases (Q13_4, p < 0.01), evaluating evidence from scientific studies relevant to patients’ health problems (Q13_5, p = < 0.01), listing all possible treatment options for the patient (Q13_6, p < 0.0001), selecting the most appropriate medication from all possible options (Q13_7, p < 0.001) and justifying their treatment decisions with evidence and reasoning (Q13_8, p < 0.0001).

**Table 3:** T-test results evaluating student responses from the established degree and the transformed degree

Factor	Question no.	Wording	Unit, Scale a	Mean of established degree	Mean of transformed degree	Mean difference ± SEM	p value*
Selfefficacy in clinical activities	The following lists different patient care activities. Rate how confident you are that you can do them as of now. Rate your degree of confidence by recording a number from 0 to 100 using the scale given below:		100-point scale				
	Q11_1	In a community pharmacy, diagnose and make recommendations for uncomplicated illnesses (e.g., cough)		83.55	83.42	-0.13 ± 1.66	0.94
	Q11_2	Conduct a detailed and systematic medication history		77.10	84.38	7.28 ± 1.61	< 0.00001
	Q11_3	In a hospital, reconcile the medication history with the previous medication list		77.49	80.96	3.47 ± 1.60	0.03
	Q11_4	In a hospital, establish an accurate medication list for a patient at discharge		76.97	78.63	1.66 ± 1.57	0.29
	Q11_5	Counsel on the use of commonly prescribed medications (e.g., statins)		80.52	83.77	3.23 ± 1.57	0.04
	Q11_6	Counsel on the use of devices (i.e., inhalers, eye drops, nasal sprays)		77.92	83.84	5.91 ± 1.73	< 0.001
	Q11_7	Write succinct yet comprehensive clinical notes (i.e., SOAP notes)		71.00	76.37	5.37 ± 1.62	< 0.01
	Q12_1	Alter patient interactions based on various patient circumstances (e.g., emotions, events)		73.81	75.62	1.81 ± 1.64	0.27
	Q12_2	Adhere to the social and ethical standards of the pharmacy profession		82.64	83.49	0.85 ± 1.62	0.60
	Q12_3	Explain medications to the patient so that they understand		82.12	83.90	1.78 ± 1.33	0.18
	Q12_4	Reflect on your performance to accurately identify what went well and what could be improved		80.22	82.81	2.59 ± 1.56	0.10
	Q12_5	Work effectively with other health care professionals to provide high-quality patient care		78.66	78.15	-0.51 ± 1.70	0.77
	Q12_6	Write specific, measurable, actionable, relevant, and timely goals/plans to improve learning and practice		74.59	77.40	2.81 ± 1.77	0.11
	Q12_7	Write clearly, concisely, and virtually error-free for a professional audience		75.37	77.47	2.10 ± 1.70	0.22
	Q13_1	Assess whether medication doses are appropriate		75.89	83.97	8.09 ± 1.57	< 0.000001
	Q13_2	Assess whether labs/tests are in-range or out-of-range		73.64	80.07	6.43 ± 1.71	< 0.001
	Q13_3	Identify medication related problems		73.12	80.27	7.16 ± 1.43	< 0.000001
	Q13_4	Use resource databases (e.g., AMH, MIMS) to answer questions and support recommendations		87.49	91.37	3.88 ± 1.26	< 0.01
	Q13_5	Evaluate evidence from scientific studies relevant to patients’ health problems		73.81	78.70	4.89 ± 1.65	< 0.01
	Q13_6	List all possible treatment options for the patient		74.07	80.14	6.07 ± 1.49	< 0.0001
	Q13_7	Select the most appropriate medication from all possible options		74.59	80.14	5.55 ± 1.45	< 0.001
	Q13_8	Justify your treatment decisions with evidence and reasoning		77.23	83.01	5.78 ± 1.46	< 0.0001
Weekday time management	Over this past semester, how many hours per day, on average, during a WEEKDAY did you spend on the following?		Hours				
	Q16_1	Sleeping		6.95	7.30	0.34 ± 0.13	< 0.01
	Q16_2	Studying/Revising		4.58	4.47	-0.11 ± 0.30	0.67
	Q16_3	Attending lectures / workshops		3.41	4.45	1.04 ± 0.19	< 0.000001
	Q16_4	Working for pay		4.28	2.95	-1.34 ± 0.28	< 0.00001
	Q16_5	Viewing media or social media (e.g., Netflix, Instagram)		3.59	3.34	-0.25 ± 0.24	0.29
	Q16_6	Exercising		1.43	0.90	-0.52 ± 0.13	< 0.001
	Q16_7	Engaging in social activities (e.g., friends, family)		2.97	2.04	-0.94 ± 0.20	< 0.00001
Weekend time management	Over this past semester, how many hours per day, on average, during a WEEKEND did you spend on the following?		Hours				
	Q17_1	Sleeping		7.68	7.94	0.26 ± 0.14	0.01
	Q17_2	Studying/Revising		4.82	4.72	-0.10 ± 0.25	0.69
	Q17_3	Attending lectures / workshops		0.74	0.27	-0.47 ± 0.14	< 0.001
	Q17_4	Working for pay		5.74	5.43	-0.31 ± 0.33	0.34
	Q17_5	Viewing media or social media (e.g., Netflix, Instagram)		3.89	3.65	-0.25 ± 0.23	0.28
	Q17_6	Exercising		1.47	1.03	-0.43 ± 0.15	< 0.01
	Q17_7	Engaging in social activities (e.g., friends, family)		3.93	2.72	-1.20 ± 0.22	< 0.000001
Satisfaction in education	Please indicate the extent to which you agree with the following statements:		%				
	Q18_1	Overall, I am satisfied with the quality of my pharmacy education		67.61	80.00	12.39 ± 2.0	< 0.000001
	Q18_2	My pharmacy school has done a good job of fostering and nurturing my development as a person		66.59	78.48	11.89 ± 2.18	< 0.000001
	Q18_3	My pharmacy school has done a good job of fostering and nurturing my development as a future pharmacist		69.12	82.76	13.64 ± 2.00	< 0.000001
Usefulness of course activities	For the following educational activities, rate how much you agree with how useful the activity was for your development as a future pharmacist		Scale 1–7 ^a^				
	Q19_1	Community placements		5.15	5.70	0.50 ± 0.17	< 0.01
	Q19_2	Hospital placements		6.22	6.46	0.24 ± 0.10	0.01
	Q19_3	Campus-based lectures		5.32	5.85	0.53 ± 0.12	< 0.00001
	Q19_4	Campus-based online learning modules		4.84	5.97	1.14 ± 0.13	< 0.000001
	Q19_5	Campus-based workshops		5.63	6.01	0.37 ± 0.11	< 0.001
	Q19_6	OSCEs		5.66	5.88	0.22 ± 0.13	0.09
Self-efficacy in career choice	Q20	If you could revisit your university choice, would you choose to attend Monash again?	Scale 1–5 ^a^	3.93	4.23	0.29 ± 0.11	< 0.01
	Q22	If you could revisit your career choice, would you choose to attend pharmacy school again?		3.24	3.80	0.55 ± 0.13	< 0.0001

^a^ items coded such that higher scores indicate higher satisfaction

*p-value related to independent t- test

Regarding weekday time management, students in the transformed degree reported sleeping more (Q16_1, p < 0.01), attending more lectures/workshops (Q16_3, p < 0.000001), exercised less (Q16_6, p < 0.001) and spent less time engaging in social activities (Q16_7, p < 0.00001) in comparison to students in the established degree (Table 3).

Students in the transformed degree also reported spending more time sleeping on weekends (Q17_1, p = 0.01) compared to students in the established degree (Table 3). The time students spent on studying and revising, however, remained consistent between the established degree and the transformed degree; 4.82 h and 4.72 h respectively.

In comparison to the established degree students, students in the transformed degree reported being more satisfied with all aspects of their education (Table 3). This includes the quality of their education (Q18_1, p < 0.000001), their development as a person as part of their education (18_2, p < 0.000001) and their development as a future pharmacist (Q18_3, p < 0.000001).

A number of course activities were evaluated as part of the survey. Results showed that students in the transformed degree found most of their course activities statistically more useful than students in the established degree (Table 3). These course activities include community placements (Q19_1, p < 0.01), hospital placements (Q19_2, p = 0.01), campus-based lectures (Q19_3, p < 0.00001), campus-based online learning modules (Q19_4, p < 0.000001), and campus-based workshops (Q19_5, p < 0.001). The *t-*test results also showed that the only course activity that the transformed degree students compared to the established degree students did not find more useful were OSCEs (Q19_6, p = 0.09).

In comparison to students in the established degree, students in the transformed degree statistically had a greater self-efficacy in their career choice. These results showed that these students from the transformed pharmacy degree were more likely to choose to both attend Monash again (Q20, p < 0.01) and pharmacy school (Q22, p < 0.0001) than established degree students.

## Discussion

The main focus of the present study was to examine the association between student characteristics, experiences and their preparedness as pharmacists as an outcome of complete degree transformation. The empirical results from the end-of-course survey showed that students who completed the transformed pharmacy curriculum reported having positive experiences throughout their degree and felt more prepared for practice as pharmacists in comparison to students who completed the established degree. Given that demographic characteristics (Table 1) remained consistent between the two cohorts, these positive findings can be associated with the design and implementation of the transformed degree.

According to self-efficacy theory and research, self-efficacy is a strong and consistent predictor for future performance. [20] Self-reported self-efficacy in several clinical activities was observed as being statistically higher in the transformed degree students compared to those in the established degree. Typically, clinical competency and self-efficacy have been shown to be developed through opportunities to rehearse, experience, observe, reflect and receive constructive feedback [25–28], thereby developing mastery of skills and experiences [20]. Hence, a factor positively impacting the development of students’ self-efficacy in clinical skills in the transformed degree is the earlier exposure to, and more extensive experiential learning placements that enable students to better practice and develop their core pharmacist clinical skills. Increased self-efficacy does not necessarily translate to improved pharmacy practice. However, we have previously shown that pharmacy preceptors were more satisfied or impressed with transformed pharmacy degree students’ ability to perform clinical activities such as history-taking, counselling, and completing medication management plans compared with established degree students [3], suggesting that the increased self-efficacy of transformed degree students does translate to improved clinical outcomes. Given that graduate hospital interns from other institutions in Australia have felt inadequately prepared for aspects of their role such as medication history taking and medication reconciliation [29], the results from the Monash end-of-degree survey are encouraging as they indicate students from the transformed degree feel they are well prepared for hospital internships (i.e., year-long hospital-based programs). Transformed degree students also reported being more motivated to complete a clinical internship in hospital pharmacy. Earlier exposure experiential placements, introduction of entrustable professional activities (EPAs) [2, 3], integration of research and interprofessional training [3], combined with regular assessment of various clinical and practical skills through OSCEs [3] and regular feedback through ‘close the loop’ lectures and regular cycles of student reflection on the development of skills, through a structured skills coaching program enabled students to develop greater confidence in performing clinical activities. This suggests that transformed degree graduates were better prepared for hospital internships having been exposed to working environments and real-life scenarios throughout their degree, and hence reported being more confident in their career choice than established degree students (Table 3).

Students from the transformed degree had higher utility-value ratings for placements, workshops, and lectures than the established degree students. This result provides one source of evidence that the changes made to the curriculum were more aligned with what students require to become future pharmacists. According to expectancy-value theory and research, [30] when students believe an activity is useful, then students are more likely to persist, learn, and engage in deeper learning activities (i.e., not just go through the motions). Also, when students value an activity, theoretically, this indirectly predicts their performance and future decisions including career choice and career aspirations. [22].

Another positive outcome of the transformed degree was changes in student’s week day time management. Time management skills are an array of behavioral skills important in a student’s organization of study and course load [31]. Given that lecture attendance is associated with better academic performance [32, 33], it was pleasing to see that compared to the established degree students, students from the transformed degree spent more time attending lectures and workshops. Furthermore, the transformed degree students, without having to spend additional time studying and revising compared to the established degree students, also reported greater self-efficacy in a number of clinical activities (Table 2). These improvements in time management may reflect enhanced organizational skills and professional development as future pharmacists, as it is important to note that a conscious decision was made when developing the transformed degree to ensure that in their fourth year, students develop greater autonomy. This is a promising outcome especially since feelings of inadequate time to complete all work is a significant stressor and can be a cause of burnout in healthcare professionals [34, 35].

Transformed degree students allocated adequate time for sleep in comparison to established degree students. Obtaining more than 7 h of sleep per day for adults is essential for optimum health and well-being [29]. It is hypothesized that longer sleep duration would lead to better academic performance based on the scientific foundation related to the effect of sleep on cognitive performance. Sleep has an integral role in learning and memory consolidation [36], whereby the duration of sleep the night prior to an examination has been found to be associated with academic performance as measured by course grades and semester grade point average (GPA). Although the end of degree survey did not assess academic performance for examination results, this would be an important point to consider during future appraisals of the transformed degree.

One particular difference between the established and transformed degree was the implementation of the flipped classroom approach, in which the learner is first exposed to online content, subsequently reinforced during face-to face sessions [37]. Although this approach was initially commenced in all courses and activities during the final year of the established degree, it was implemented throughout all year levels of the transformed degree [1]. Students experienced ‘flipped’ classroom active learning during campus-based lectures, campus-based online learning modules, and campus-based workshops. Given that transformed degree students found most of their course activities more useful than their established degree counterparts (Table 3), this is a favorable result, as blended learning in pharmacy education has been shown to significantly improve the learning outcomes of students, as it incorporates face-to-face and online components allowing students to work at their own pace and time, whilst also allowing for class meaningful discussions of the material [37]. In addition, the results of the survey also suggest that the design and implementation of online modules by the faculty improved over the years since the commencement of the ‘flipped’ approach. Furthermore, the innovative design of the transformed degree encompassing blended learning with a focus on building work-place ready core pharmacist skills produced students who were more satisfied with their education in comparison to the students who completed the established degree (Table 3), indicating that the transformed degree was academically well designed and offered students an enjoyable university experience.

### Limitations

This study was designed to provide a cross-sectional view of student views and experiences upon completion of the transformed Monash pharmacy degree in comparison to the previous established degree. Although the study did not attempt to account for any differences at baseline between the two cohorts, as a post-test study it examined the outcomes of complete degree transformation from a student perspective, which is certainly a positive aspect of this research.

The novelty and innovativeness of the transformed degree could have potentially influenced students’ perception of their experience and their evaluation of the degree’s effectiveness in preparing them for practice. This may have resulted in a positive bias in their responses compared to students from the established curriculum. Furthermore, students from the established curriculum may have expressed dissatisfaction or negative attitudes towards not having the chance to take advantage of the new curriculum, which could have impacted their responses to the survey. It is essential to acknowledge these potential biases when interpreting the results of this study.

The dissemination of the survey differed between the two cohorts because of the impact of the COVID-19 pandemic and may have affected the response rate and outcomes in 2020. However, as with the design and implementation of the transformed degree, the faculty worked with comradery to swiftly convert to online modalities of teaching and learning in response to the COVID-19 pandemic to ensure continued delivery of the pharmacy curriculum and the training of students [38]. To facilitate this transition, all students and staff received training and resources on utilizing the virtual resources that were implemented [38]. Furthermore, a conscious decision was made when developing the transformed degree to ensure that in their fourth-year students develop greater autonomy through a greater focus on online learning [1], as such, the impact of COVID-19 on transformed degree students may not have been as consequential on their education as those belonging to other cohorts and institutions. However, it is worth noting that students in the transformed degree program, particularly those in Melbourne, experienced extensive lockdowns during the pandemic, which could have affected their regular behavior, such as weekday and weekend time management. As such, future research should examine the amount of time students spend on different activities and consider the potential influence of COVID-19 lockdowns.

## Conclusions

Student responses to the end of degree survey indicate that the initial group of students to complete the transformed Monash pharmacy curriculum have had positive experiences throughout their degree and felt more prepared for practice as pharmacists in comparison to students who completed the established degree. These results add value to those collected from other sources (e.g., student evaluations, assessment scores, preceptors focus groups, and other stakeholder inputs) consistent with a comprehensive quality improvement model. Furthermore, unlike previous literature that only assess changes to individual courses within a degree [4], the results from this survey demonstrate how student experiences can be effectively assessed as an outcome of complete degree transformation using an evidence based transparent survey design specific to pharmacy education. Although the results from this study have shown favorable outcomes in support of the transformed Monash pharmacy degree, end of course survey evaluations from students from succeeding years are necessary to determine if these outcomes have been sustained. Specific information about the impact of COVID-19 restrictions on student and teacher experiences would also be valuable. In addition, surveying students as they progress through the degree or even after they have worked in their professional careers for a couple of years may also indicate the extent to which their self-efficacy in clinical activities, weekday time management, weekend time management, satisfaction in education, usefulness of course activities and self-efficacy in career choice changes throughout their university journey.

## Electronic supplementary material

Below is the link to the electronic supplementary material.

Supplementary Material 1

## Data Availability

The datasets used and/or analysed during the current study are available from the corresponding author on reasonable request.
