# Supplementary material for: Effect of a curriculum transformation on pharmacy student self-efficacy, self-reported activities, and satisfaction in degree and career choice
Source: BMC Med Educ. 2023 May 2;23:304. doi: 10.1186/s12909-023-04280-7 (PMC10152417; doi:10.1186/s12909-023-04280-7)
Supplement: Supplementary file 1 — Supplementary Material 1 [file 12909_2023_4280_MOESM1_ESM.docx]

Appendix 1: End of course survey

| Factor | Unit, Scale ^a^ | Question no. | Wording |
| --- | --- | --- | --- |
| Demographic information | Nil | Q1 | What is your first and last (surname) name? |
|  |  | Q2 | What is your student ID? |
|  |  | Q3 | If you win the raffle, what email address should we send the $50 gift card to? |
|  |  | Q4 | What country were you born in? |
|  |  | Q5 | What was the first language you learned as a child? |
|  |  | Q6 | What language do you speak at home? |
|  |  | Q7 | Are you a graduate entry student? (yes/no) |
|  |  | Q8 | What are your plans for next year? (community internship/ hospital internship/ other/ unknown) |
|  |  | Q9 | What city and country is your internship in? |
|  |  | Q10 | Describe what makes a “good” pharmacist, in your opinion. Please write at least 4-5 sentences |
| Confidence in clinical activities | % | Q11_1 | The following lists different patient care activities. Rate how confident you are that you can do them as of now. Rate your degree of confidence by recording a number from 0 to 100 using the scale given below: - In a community pharmacy, diagnose and make recommendations for uncomplicated illnesses (e.g., cough) |
|  |  | Q11_2 | The following lists different patient care activities. Rate how confident you are that you can do them as of now. Rate your degree of confidence by recording a number from 0 to 100 using the scale given below: - Conduct a detailed and systematic medication history |
|  |  | Q11_3 | The following lists different patient care activities. Rate how confident you are that you can do them as of now. Rate your degree of confidence by recording a number from 0 to 100 using the scale given below: - In a hospital, reconcile the medication history with the previous medication list |
|  |  | Q11_4 | The following lists different patient care activities. Rate how confident you are that you can do them as of now. Rate your degree of confidence by recording a number from 0 to 100 using the scale given below: - In a hospital, establish an accurate medication list for a patient at discharge |
|  |  | Q11_5 | The following lists different patient care activities. Rate how confident you are that you can do them as of now. Rate your degree of confidence by recording a number from 0 to 100 using the scale given below: - Counsel on the use of commonly prescribed medications (e.g., statins) |
|  |  | Q11_6 | The following lists different patient care activities. Rate how confident you are that you can do them as of now. Rate your degree of confidence by recording a number from 0 to 100 using the scale given below: - Counsel on the use of devices (i.e., inhalers, eye drops, nasal sprays) |
|  |  | Q11_7 | The following lists different patient care activities. Rate how confident you are that you can do them as of now. Rate your degree of confidence by recording a number from 0 to 100 using the scale given below: - Write succinct yet comprehensive clinical notes (i.e., SOAP notes) |
|  |  | Q12_1 | The following lists different clinical skills. Rate how confident you are that you can do them as of now. Rate your degree of confidence by recording a number from 0 to 100 using the scale given below: - Alter patient interactions based on various patient circumstances (e.g., emotions, events) |
|  |  | Q12_2 | The following lists different clinical skills. Rate how confident you are that you can do them as of now. Rate your degree of confidence by recording a number from 0 to 100 using the scale given below: - Adhere to the social and ethical standards of the pharmacy profession |
|  |  | Q12_3 | The following lists different clinical skills. Rate how confident you are that you can do them as of now. Rate your degree of confidence by recording a number from 0 to 100 using the scale given below: - Explain medications to the patient so that they understand |
|  |  | Q12_4 | The following lists different clinical skills. Rate how confident you are that you can do them as of now. Rate your degree of confidence by recording a number from 0 to 100 using the scale given below: - Reflect on your performance to accurately identify what went well and what could be improved |
|  |  | Q12_5 | The following lists different clinical skills. Rate how confident you are that you can do them as of now. Rate your degree of confidence by recording a number from 0 to 100 using the scale given below: - Work effectively with other health care professionals to provide high-quality patient care |
|  |  | Q12_6 | The following lists different clinical skills. Rate how confident you are that you can do them as of now. Rate your degree of confidence by recording a number from 0 to 100 using the scale given below: - Write specific, measurable, actionable, relevant, and timely goals/plans to improve learning and practice |
|  |  | Q12_7 | The following lists different clinical skills. Rate how confident you are that you can do them as of now. Rate your degree of confidence by recording a number from 0 to 100 using the scale given below: - Write clearly, concisely, and virtually error-free for a professional audience |
|  |  | Q13_1 | The following lists different activities performed while reviewing a patient's record. Rate how confident you are that you can do them as of now. Rate your degree of confidence by recording a number from 0 to 100 using the scale given below: - Assess whether medication doses are appropriate |
|  |  | Q13_2 | The following lists different activities performed while reviewing a patient's record. Rate how confident you are that you can do them as of now. Rate your degree of confidence by recording a number from 0 to 100 using the scale given below: - Assess whether labs/tests are in-range or out-of-range |
|  |  | Q13_3 | The following lists different activities performed while reviewing a patient's record. Rate how confident you are that you can do them as of now. Rate your degree of confidence by recording a number from 0 to 100 using the scale given below: - Identify medication related problems |
|  |  | Q13_4 | The following lists different activities performed while reviewing a patient's record. Rate how confident you are that you can do them as of now. Rate your degree of confidence by recording a number from 0 to 100 using the scale given below: - Use resource databases (e.g., AMH, MIMS) to answer questions and support recommendations |
|  |  | Q13_5 | The following lists different activities performed while reviewing a patient's record. Rate how confident you are that you can do them as of now. Rate your degree of confidence by recording a number from 0 to 100 using the scale given below: - Evaluate evidence from scientific studies relevant to patients' health problems |
|  |  | Q13_6 | The following lists different activities performed while reviewing a patient's record. Rate how confident you are that you can do them as of now. Rate your degree of confidence by recording a number from 0 to 100 using the scale given below: - List all possible treatment options for the patient |
|  |  | Q13_7 | The following lists different activities performed while reviewing a patient's record. Rate how confident you are that you can do them as of now. Rate your degree of confidence by recording a number from 0 to 100 using the scale given below: - Select the most appropriate medication from all possible options |
|  |  | Q13_8 | The following lists different activities performed while reviewing a patient's record. Rate how confident you are that you can do them as of now. Rate your degree of confidence by recording a number from 0 to 100 using the scale given below: - Justify your treatment decisions with evidence and reasoning |
| Future career | Nil | Q14 | Please indicate the setting in which you plan to work after the completion of your internship (Large city or suburb of a large city (population of 500,000 or more)/ City of a moderate size (population 50,000 to 500,000)/ City of a moderate size (population 50,000 to 500,000)/ City of a moderate size (population 50,000 to 500,000)/ City of a moderate size (population 50,000 to 500,000) |
|  |  | Q15 | In which of the following activities do you plan to participate during your career? (notes can have multiple answers)  (Research/ administration leadership/ further education/ professional pharmacy organisation membership/ pharmacy school faculty/ teaching pharmacy student during placements/ part-time teaching students in lectures, workshops, or skills coaching) |
| Weekday time management | Hours | Q16_1 | Over this past semester, how many hours per day, on average, during a WEEKDAY did you spend on the following? - Sleeping |
|  |  | Q16_2 | Over this past semester, how many hours per day, on average, during a WEEKDAY did you spend on the following? - Studying/Revising |
|  |  | Q16_3 | Over this past semester, how many hours per day, on average, during a WEEKDAY did you spend on the following? - Attending lectures / workshops |
|  |  | Q16_4 | Over this past semester, how many hours per day, on average, during a WEEKDAY did you spend on the following? - Working for pay |
|  |  | Q16_5 | Over this past semester, how many hours per day, on average, during a WEEKDAY did you spend on the following? - Viewing media or social media (e.g., Netflix, Instagram) |
|  |  | Q16_6 | Over this past semester, how many hours per day, on average, during a WEEKDAY did you spend on the following? - Exercising |
|  |  | Q16_7 | Over this past semester, how many hours per day, on average, during a WEEKDAY did you spend on the following? - Engaging in social activities (e.g., friends, family) |
| Weekend time management | Hours | Q17_1 | Over this past semester, how many hours per day, on average, during a WEEKEND did you spend on the following? - Sleeping |
|  |  | Q17_2 | Over this past semester, how many hours per day, on average, during a WEEKEND did you spend on the following? - Studying/Revising |
|  |  | Q17_3 | Over this past semester, how many hours per day, on average, during a WEEKEND did you spend on the following? - Attending lectures / workshops |
|  |  | Q17_4 | Over this past semester, how many hours per day, on average, during a WEEKEND did you spend on the following? - Working for pay |
|  |  | Q17_5 | Over this past semester, how many hours per day, on average, during a WEEKEND did you spend on the following? - Viewing media or social media (e.g., Netflix, Instagram) |
|  |  | Q17_6 | Over this past semester, how many hours per day, on average, during a WEEKEND did you spend on the following? - Viewing media or social media (e.g., Netflix, Instagram) |
|  |  | Q17_7 | Over this past semester, how many hours per day, on average, during a WEEKEND did you spend on the following? - Engaging in social activities (e.g., friends, family) |
| Satisfaction in education | % | Q18_1 | Please indicate the extent to which you agree with the following statements: - Overall, I am satisfied with the quality of my pharmacy education |
|  |  | Q18_2 | Please indicate the extent to which you agree with the following statements: - My pharmacy school has done a good job of fostering and nurturing my development as a person |
|  |  | Q18_3 | Please indicate the extent to which you agree with the following statements: - My pharmacy school has done a good job of fostering and nurturing my development as a future pharmacist |
| Usefulness of course activities | (Scale 1-7) ^a^  1.00= "strongly disagree"  2.00= "disagree"  3.00="somewhat disagree"  4.00="neither agree nor disagree"  5.00=somewhat agree"  6.00="agree"  7.00="strongly agree" | Q19_1 | For the following educational activities, rate how much you agree with how useful the activity was for your development as a future pharmacist - Community placements |
|  |  | Q19_2 | For the following educational activities, rate how much you agree with how useful the activity was for your development as a future pharmacist - Hospital placements |
|  |  | Q19_3 | For the following educational activities, rate how much you agree with how useful the activity was for your development as a future pharmacist - Campus-based lectures |
|  |  | Q19_4 | For the following educational activities, rate how much you agree with how useful the activity was for your development as a future pharmacist - Campus-based online learning modules |
|  |  | Q19_5 | For the following educational activities, rate how much you agree with how useful the activity was for your development as a future pharmacist - Campus-based workshops |
|  |  | Q19_6 | For the following educational activities, rate how much you agree with how useful the activity was for your development as a future pharmacist - OSCEs |
| Confidence in career choice | (Scale 1-5) ^a^  1 ="no'  2 ='probably no'  3 "='neutral'  4 ="probably yes"  5 ="yes" | Q20 | If you could revisit your university choice, would you choose to attend Monash again? |
|  |  | Q22 | If you could revisit your career choice, would you choose to attend pharmacy school again? |
